# The human microbiome and its link in prostate cancer risk and pathogenesis

**DOI:** 10.1186/s13027-020-00319-2

**Published:** 2020-08-31

**Authors:** Paul Katongole, Obondo J. Sande, Moses Joloba, Steven J. Reynolds, Nixon Niyonzima

**Affiliations:** 1 Department of Medical Microbiology, College of Health Sciences Makerere University, Kampala, Uganda; 2 Department of Medical Biochemistry, College of Health Sciences Makerere University, Kampala, Uganda; 3 Department of Immunology and Molecular biology, College of Health Sciences Makerere University, Kampala, Uganda; 4 Division of Intramural Research, National Institute of Allergy and Infectious Diseases, National Institutes of Health, Bethesda, MD USA; 5 Uganda Cancer Institute, Kampala, Uganda

**Keywords:** Prostate cancer, Microbiota, Microbiome, Gut microbiome, And urinary microbiome

## Abstract

There is growing evidence of the microbiome’s role in human health and disease since the human microbiome project. The microbiome plays a vital role in influencing cancer risk and pathogenesis. Several studies indicate microbial pathogens to account for over 15–20% of all cancers. Furthermore, the interaction of the microbiota, especially the gut microbiota in influencing response to chemotherapy, immunotherapy, and radiotherapy remains an area of active research. Certain microbial species have been linked to the improved clinical outcome when on different cancer therapies. The recent discovery of the urinary microbiome has enabled the study to understand its connection to genitourinary malignancies, especially prostate cancer. Prostate cancer is the second most common cancer in males worldwide. Therefore research into understanding the factors and mechanisms associated with prostate cancer etiology, pathogenesis, and disease progression is of utmost importance. In this review, we explore the current literature concerning the link between the gut and urinary microbiome and prostate cancer risk and pathogenesis.

## Introduction

The human microbiota plays a vital role in many life processes, both in health and disease [1, 2]. The microbiota is defined as a group of microorganisms; bacteria, viruses, fungi, and archaea living in a host, whereas the microbiome is a collection of genes and genomes of the microbiota and their environment [2]. The host and the gut microbiome exist in a symbiotic equilibrium state [3]. Microbial dysbiosis occurs when this state of equilibrium is distorted [4, 5] and can be caused by stressors such as disease, age, diet, smoking, and many other environmental factors. Understanding the mechanisms of this unique niche and balance is critical in understanding the microbiome’s role in many disease processes, including cancer pathogenesis [6, 7]. The mechanisms by which the microbiota can alter cancer risk and progression are primarily attributed to immune system modulation through mediators of chronic inflammation. These can switch on oncogenic metabolites such as reactive oxygen and nitrogen species that can lead to DNA damage, triggering tumorigenesis [8].

Prostate cancer is the second most common cancer among men worldwide. In 2018, the Global Cancer Observatory (Globocan) report indicated 1,276,106 new prostate cancer cases, with approximately 358,989 deaths from the same disease [9]. The management of prostate cancer has seen a lot of advancement from chemotherapy, especially using androgen deprivation therapy, surgery for low-grade tumors, hormonal therapy, and radiotherapy [10]. However, there has remained a gap, especially in the management of advanced prostate cancer, with many patients developing resistance to primary chemotherapeutic agents, especially in castration-resistant prostate cancer (CRPC) [11]. With the introduction of new treatment options such as immunotherapy and the advancement in sequencing technologies, e.g., next-generation sequencing (NGS) and metagenomics, there is hope that the microbiome, a critical factor in the regulation of immune system could be a promising future therapeutic option in the management of cancers including prostate cancer [12]. This is, however, still an active area of research with many clinical trials underway. Therefore, it is essential to understand the link between the microbiome and different cancers, including prostate cancer.

Several studies have indicated that the microbiota can influence prostate tumorigenesis through direct or indirect mechanisms [13, 14]. Under direct mechanisms, Prostate cancer is associated with chronic inflammatory urinary tract conditions such as: Chronic prostatitis and Benign Prostatic Hypertrophy (BPH), among others [15]. Under indirect mechanisms, the gut microbiota is said to influence metabolic processes and systemic inflammation that tend to trigger prostate tumorigenesis. In this review, we explore the current literature regarding microbiome effects on prostate cancer risk and pathogenesis.

### Human microbiota and cancer

There is an established link between the microbiome and cancer risk and pathogenesis [16, 17]. The human microbiota found in many different organs comprises a heterogeneous population of microorganisms, namely bacteria, fungi, and viruses that maintain the host’s homeostasis and are continually being exposed to varying stimuli throughout the lifespan of the host [18]. The microbiota composition is dependent on genetic and environmental factors, including diet, geographic location, toxin/carcinogen exposure, hormones, and antibiotics, among other exposures [19, 20]. It is hypothesized that the microbiota is a missing link in many cancer pathogeneses [21, 22]. The gut microbiota, the most diverse and highly studied group, performs a multitude of functions, including the metabolism of different dietary compounds, protection against pathogenic bacteria colonization, and maintaining the host inflammatory equilibrium, among other functions [3]. The link between the gut microbiota and cancer is bidirectional; cancer can alter microbiota’s composition, whereas microbiota can affect the progression or response of cancers [13]. There are several mechanisms by which microbiota is believed to contribute to tumorigenesis. These include; genotypic integration, chronic inflammation, genotoxicity, and immune modulation [23, 24].

Genomic integration is a virulence mechanism by most oncogenic viruses. The virus inserts its DNA into the host genome and hence partly controlling the host’s cellular replication. The classic example is HPV (Human papillomavirus) strains HPV 16 and HPV 18, which are the leading cause of cervical cancer. HPV 16/18 insert oncogenes E6 and E7 into the host genome, which silences tumor suppressor genes p53 and pRB leading to the uncontrolled cellular replication hence carcinogenesis [25, 26].

Inflammation is a fundamental feature of carcinogenesis irrespective of the etiological agent and is the primary oncogenic mechanism underlying several well-defined, causal, and microbial associations with cancer [24, 26]. Different microbial virulence factors can induce chronic inflammation in the host tissue cells, triggering cellular proliferation [27]. Uncontrolled cellular proliferation can trigger apoptosis, which can result in tumorigenesis. Chronic inflammation is hypothesized to be a trigger of prostate carcinogenesis, as evidenced by inflammatory cells in the prostate microenvironment [28]. Prostatic infections coupled with urinary tract infections provide a crucial niche for repeated inflammatory exposure to the prostate microenvironment leading to prostate cancer precursor lesions termed proliferative inflammatory atrophy [15]. Proliferative inflammatory atrophy of the prostatic epithelial cells is an intermediate phenotype prone to genomic and epigenetic alterations leading to prostatic intraepithelial neoplasia and prostate cancer [28]. However, inflammation can also trigger oxidative stress through the generation of reactive oxygen and nitrogen species that induce mutagenesis leading to prostate carcinogenesis [28]. Hence, current evidence suggests that inflammation and atrophy are involved in prostate carcinogenesis. The studies also continue to indicate that the microbiome plays a role in establishing an inflammatory prostate microenvironment that promotes prostate cancer development and progression.

Genotoxicity is a process by which cellular DNA damage happens through breaks, deletions, re-arrangements, or insertions [29]. Genotoxicity can lead to cellular death or lead to carcinogenesis by switching off tumor suppressor genes (p53 and pRB) [30]. Cytolethal distending toxin (CDT) and colibactin are genotoxins produced by microorganisms in the family Enterobacteriaceae, especially *Escherichia coli*. These induce double-strand DNA breaks leading to carcinogenesis.

Studies have shown that the disruption of the gut microbiome immune system cross-talk that maintains an anti-cancer immune surveillance system can trigger tumorigenesis [31, 32].

Therefore the current evidence indicates the microbiome is a potential therapeutic option in managing several cancers, and several clinical trials are underway to establish its clinical utility.

### Microbiome in prostate tissue microenvironment

Several studies have demonstrated the existence of bacteria and viruses in normal and cancerous prostate tissues. A study by Cohen R et al. 2005, examined 34 men who had undergone radical prostatectomy and later culture, and histological diagnosis was made on whole sections of prostatic tissues. *Propionibacterium acnes spp was* the most predominant bacteria found on culture with 35% frequency, and its presence was significantly(*p*-value = 0.007) associated with prostate tissue inflammation [33].

A similar study by Sfanos KS et al., 2008, carried out 16S rDNA gene sequencing of bacterial DNA from a series of 170 prostatectomy tissue core samples from 30 cancer patients. Over 83 species of bacteria were found in prostate cancer tissues. In this study, culturing the Prostate, core tissues didn’t yield any bacterial species, and this could be due to the presence of un-culturable species. The study found no significant association between the presence of particular species of bacteria and histologic evidence of acute or chronic inflammation [34].

More recently, Yow et al. 2017**,** performed 16S rDNA next-generation sequencing and total RNA sequencing on cancerous regions and matched benign tissue regions from 20 aggressive prostate cancer patients. Upon 16S rDNA sequencing, the study identified family *Enterobacteriaceae,* specifically the genus *Escherichia and also Propionibacterium acnes* were found with the highest relative abundance of 95%. RNA sequencing identified endogenous retroviral sequences in both malignant and benign tissue datasets, but no other known viral sequences were identified [35]. The study did not, however, have negative controls for DNA extraction and sequencing pipelines.

Cavarretta et al., 2017 investigated microbial presence in the tumor, peri-tumoral, and non-tumor areas of prostate tissues using 16S rDNA sequencing directed to the V3–V5 hypervariable regions. *Propionibacterium spp* was the most predominant bacterial genus found in all areas of the tumor. Beta diversity was not significantly different between areas, but individual bacterial species were significantly and differentially abundant in some areas. Tumor and the peri-tumoral regions had a similarly higher relative abundance of *Staphylococcus spp* compared to normal areas. In contrast, the normal areas had a higher abundance of *Streptococcus spp* than the tumor and the peri-tumoral regions [36].

A study by Feng et al. 2019, used integrated metagenomics and metatranscriptomic analysis to identify microbiota in frozen radical prostate specimens from tumor and adjacent benign tissue from 65 Chinese patients. They identified over 40 unique bacterial genera with *Pseudomonas, Escherichia, Acinetobacter, and Propionibacterium spp* being the most abundant. They did not detect any viruses. The study found no difference between tumors and benign tissue in terms of overall (alpha) bacterial diversity or group (beta) diversity, regardless of Gleason score [37].

Banerjee et al., in 2019, used microarray metagenomics analysis of formalin-fixed tissue from 50 prostate cancer patients and 15 patients with BPH and identified viral, bacterial, and fungal DNA signatures. The most predominant bacteria belonged to the following phyla; *Proteobacteria, Firmicutes, Actinobacteria, and Bacteroidetes* in order of reducing frequency. There were no differences in the microbiota signatures of cancer and BPH prostate tissues [38]. Among the viruses isolated, 41% are known tumorigenic viruses, including high-risk human papillomavirus (HPV 16&18), and human cytomegalovirus (HCMV). HPV18, Kaposi’s Sarcoma Herpesvirus (KSHV), and Polyomaviridae were found to be associated with lower Gleason scores [38].

A similar study by Miyake et al., 2019 screened 45 prostate cancer and 33 BPH patient tissue specimens for various sexually transmitted infectious agents using PCR. Out of the seven organisms that were tested for, namely; *Neisseria gonorrhoeae, Chlamydia trachomatis, Mycoplasma genitalium, Mycoplasma hyorhinis, Ureaplasma urealyticum,* HPV16, and HPV18, only *Mycoplasma genitalium* was independently associated with prostate cancer and with high Gleason scores [39]. The findings from these studies indicate that prostate cancerous tissue harbor different microbial species, which could be linked to prostatic inflammation and carcinogenesis.

### Urinary microbiome and prostate cancer

The discovery of a vast urinary microbiome’s immense diversity has changed the previous dogma of urine being sterile [40]. Previously, the microbiome’s detection in urine by culture faced a significant challenge of contamination by skin, prepuce, virginal, and rectal areas. Advancements have overcome this challenge in new, highly sensitive detection methods such as 16S RNA and DNA sequencing and shotgun metagenomics sequencing [41].

Prostate cancer has been associated with chronic urinary tract infections such as chronic prostatitis/chronic pelvic pain syndrome (CP/CPPS). Therefore [42, 43], understanding the urinary microbiome is vital in connecting dots in prostate cancer pathogenesis.

Similarly, several studies have explored the relationship between the urinary microbiome and prostate cancer [42, 44, 45]. Shrestha et al. 2018, assessed the urinary microbiome of 135 men undergoing prostate biopsy. *Corynebacterium, Staphylococcus*, and *Streptococcus* species were the most predominant in both positive and negative biopsy cases. Several other species, such as; *Streptococcus anginosus, Anaerococcus lactolyticus, Anaerococcus obesiensis, Actinobaculum schaalii, Varibaculum cambriense* and *Propionimicrobium lymphophilum* were more abundant in positive biopsy patients than negative biopsy patients [44]. There was no single microbiota spp. that was found to be significantly associated with prostate cancer. However, this study did not provide information regarding whether they did a digital rectal exam to ensure that the voided urine included expressed prostatic secretions. The other limitation of the study is that it never mentioned if the urine was a midstream clean-catch sample as this ensures minimal urethral contamination.

Yu et al. 2015 assessed microbiota in urine, seminal fluid, and expressed prostatic fluids (EPS) from men with BPH and Prostate cancer. *E. coli* detection was significantly higher in EPS and seminal fluid compared to urine, while *Enterococcus spp* detection was more common in seminal fluid. Patients with prostate cancer had significantly high *Bacteroidetes, Alphaproteobacteria, Firmicutes bacteria, Lachnospiraceae*, *Propionicimonas*, *and Sphingomonas* and *Ochrobactrum*, and a decrease in *Eubacterium* and *Defluviicoccus* compared to the BPH group [45]. This study examined the microbiota of a valuable EPS specimen, which could provide a less invasive alternative for understanding the prostate microbiome.

In a study by Alanee et al. 2018, urinary microbiota of 30 men undergoing trans-rectal prostate biopsy with high prostatic-specific antigen (PSA) levels were assessed. Urine samples were obtained, followed by a prostatic massage. Patients with prostate cancer had a high abundance of *Veillonella, Streptococcus, and Bacteroides*, and a low abundance of *Faecalibacterium, Lactobacilli, and Acinetobacter* as compared to those with BPH [46]. However, this study had a small sample size of 30 patients, which would have reduced the study’s power.

From the findings of the different studies looking at the Urinary microbiome, it remains essential to standardize the procedures and techniques for urine sample collection. This will provide a platform for comparable results [47]. We also observe that chronic inflammation, coupled with prostatic and or urinary tract infections, provides an inflammatory microenvironment that stimulates the development of prostate cancer precursor lesions that drive prostate tumorigenesis [48].

### The gut microbiota and prostate cancer

There is a paucity of knowledge on how the gut microbiome influences prostate cancer risk and pathogenesis [49, 50]. Studies have explored the gut microbiome composition that regulates the metabolism of compounds associated with increased prostate cancer risk. Other studies have, however, examined the microbiota composition in prostate cancer cases and their controls.

Studies have shown that a regular dietary composition of dairy products, red meat, and high fat is associated with increased prostate cancer risk [51, 52].

Antibiotics have been shown to induce gut microbial dysbiosis, which can propagate translocation of pathogenic bacteria, leading to chronic inflammation, an essential inducer of tumorigenesis [53, 54]. A study by Boursi et al. .2015 analyzed a retrospective dataset of 27,212 cases and 105,940 controls. It was found that prostate cancer risk increased modestly with the use of penicillin and quinolones, sulphonamides, and tetracyclines [55]. A study by Plottel et al., 2011 hypothesized that the estrobolome (enteric genes that metabolize estrogen) is associated with prostate cancer risk. Estrogen is said to activate polycyclic hydrocarbons leading to the formation of carcinogenic metabolites, e.g., radical cations that induce cellular DNA damage leading to carcinogenesis [56].

Studies have also explored how specific gut microbiota are associated with prostate cancer risk and outcomes. Liss et al. 2018, assessed the gut microbiota among 133 men undergoing trans-rectal prostate biopsy. At the species level, they found significant differences between cancer and non-cancer groups for some well-represented members, including enriched *Bacteroides and Streptococcus spp* in cancer compared with the non-cancer control group. The study did not find any significant differences in the microbial diversity between prostate cancer and controls [57].

Golombos et al. 2018 evaluated the gut microbiome of 20 men with benign prostatic hypertrophy and prostate cancer (localized/intermediate and high risk) undergoing care at a tertiary health facility. *Bacteroides massiliensis* was found to be in high relative abundance in prostate cancer cases compared to controls. *Feacalibactereium prausnitzii* and *Eubactererium rectalie* were in a higher relative abundance among controls [49]. The study found significant biological differences at gene level pathways between cases and controls.

Alanee et al. 2018 in a prospective study, assessed the gut microbiota of 30 men among men undergoing trans-rectal prostate biopsy. Upon histological diagnosis, the study observed an increased abundance of *Bacteroides spp* among patients with prostate cancer than the controls [46]. The study did not find a significant association between microbiota clustering patterns and Gleason scores of prostate cancer patients.

**Fig. 1:**
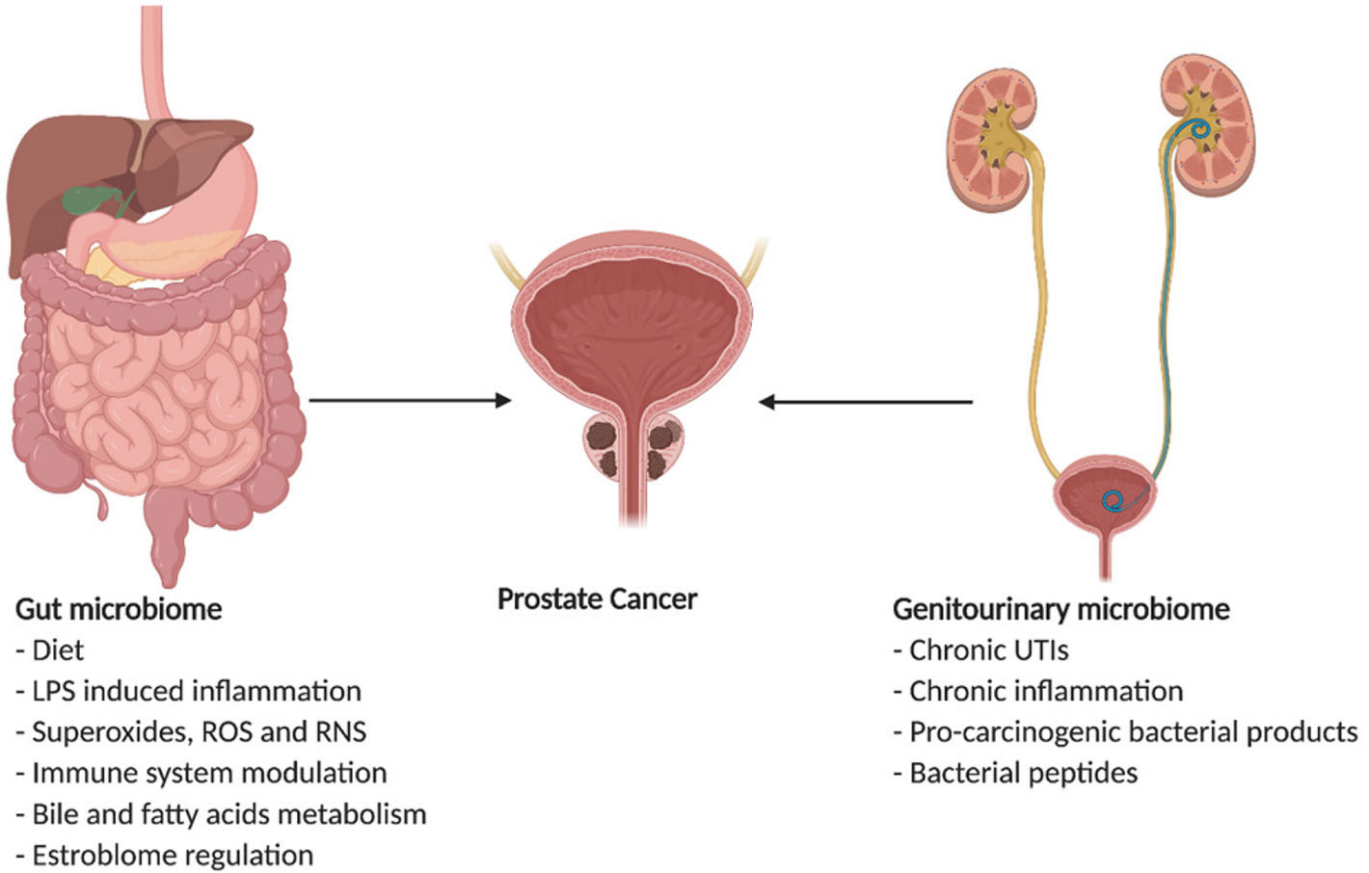
Potential mechanisms of actions of how gut and genitourinary microbiome interacts with Prostate cancer

A study by Sfanos et al. 2018, carried out a cross-sectional study where they profiled the fecal microbiota of 30 healthy male volunteers and men with different clinical states of prostate cancer (i.e., localized, biochemically recurrent, and metastatic disease) using 16S rDNA amplicon sequencing. The study reported a greater alpha diversity in those without prostate cancer than those with prostate cancer [58]. The findings from different studies indicate a plausible link between specific gut microbial species and prostate cancer risk and disease status (Fig. 1).

### Microbiota and prostate cancer treatment

There is growing evidence that gut microbiota modulates response to different drugs, including chemotherapeutic agents [59]. Alexander et al., 2017 proposed a TIMER model (Translocation, Immunomodulation, Metabolism, Enzymatic degradation, and reduced diversity). The TIMER model expounds on the mechanisms for how gut microbiota mechanistically influences chemotherapeutic agents [60].

Under microbial translocation, Viaud et al., 2013 found that cyclophosphamide caused the shortening gut intestinal wall villi, allowing microbes to cross and enter secondary lymphoid organs such as lymph nodes, tonsils, and the spleen. They hypothesized that cyclophosphamide stimulates anti-tumor immune responses of gut microbiota from lymphoid organ infiltration. The study also found out that a specific set of gram-positive bacteria (*Lactobacillus johnsonii*, *Lactobacillus murinus,* and *Enterococcus hirae*) were necessary to mediate cyclophosphamide-driven accumulation of type 17 T helper (TH17) cell and type 1 T helper (TH1) cell responses [61].

A study by Lida et al.,2013 found that intestinal microbiota in mice facilitated the immunomodulation of chemotherapeutic drugs through the regulation of immune cytokines and innate myeloid cell release and the introduction of antibiotics further reduced the anti-tumor effect of the chemotherapeutic agents [62]. The findings from these and several other studies have illustrated the active role of the microbiota in immune modulation and how this could influence the response and efficacy of different chemotherapeutic agents [59].

Different studies have indicated that bacteria in the gastrointestinal tract regulate metabolic processes such as reduction, hydrolysis, dihydroxylation, and dealkylation, which affects the efficacy of various chemotherapeutic agents [60, 63]. For reduced diversity, Montassier et al., 2015 found that fecal samples collected after chemotherapy contained a decreased abundance of *Firmicutes, Actinobacteria*, and increases in *Proteobacteria* than the patients’ samples before chemotherapy [64]. In a similar study by Vande et al. 2013, Mycoplasma *hyorhinis* was found to metabolize the prostate cancer drug Gemcitabine into an inactive metabolite, therefore decreasing the efficacy of the drug [65].

A study by Sfanos et al. 2018, hypothesized that taking oral androgen deprivation therapies (ADT), including bicalutamide, enzalutamide, and abiraterone acetate, was associated with compositional differences in the gut microbiota. **The study reported** a significant difference in alpha diversity in gut microbiota among men with prostate cancer versus those without a prostate cancer diagnosis. There were significant compositional differences in the gut microbiota of men taking ADT, including a greater abundance of species such as *Akkermansia muciniphila* and *Ruminococcaceae spp*. Furthermore, upon functional analysis, the study found an enriched representation of bacterial gene pathways involved in steroid hormone biosynthesis in the fecal microbiota of men taking oral ADT. The findings from these studies, therefore, creates a need for more research into understanding the clinical efficacy of the gut microbiota in Prostate cancer clinical management [58] (Table 1).

**Table 1:** Summary of studies on the Microbiome and Prostate cancer

Study	Samples	Findings
Cohen R et al. 2005 [33]	34 Prostate tissues cultured from men after undergoing radical prostatectomy	*Propionibacterium acnes spp was* the most predominant bacteria and significant prostate tissue inflammation was observed
Sfanos KS et al., 2008 [34]	Prostatectomy tissues from 30 men with Prostate cancer underwent 16S rDNA sequencing	83 distinct microorganisms spp. identified. There was no significant association between the presence of particular species of bacteria and histologic evidence of acute or chronic inflammation.
Yow MA et al. 2017 [35]	16S rDNA sequencing on 20 snap-frozen prostate tissue cores from ten “aggressive” prostate cancer cases	*Enterobacteriacae* member species were found common to all samples and *P. acnes* in 95% of analyzed samples.
Cavarretta I et al., 2017 [36]	Performed 16S rDNA microbiome sequencing of tumor, peri-tumor, and non-tumor tissues upon radical prostatectomy.	*Propionibacterium spp* was the most predominant bacterial genus found in all regions of the tumor. *Staphylococcus spp* was the more abundant in tumor and peri-tumor areas as compared to normal tissue areas.
Feng Y et al., 2019 [37]	Metagenomics and meta-transcriptomic analysis to identify microbiota in frozen radical prostate specimens from tumor and adjacent benign tissue from 65 Chinese patients	40 unique bacterial genera were identified. *Pseudomonas, Escherichia, Acinetobacter, and Propionibacterium spp were* the most abundant spp. respectively.
Banerjee S et al.,2019 [38]	Microarray metagenomics analysis of formalin-fixed tissue from 50 prostate cancer patients and 15 patients with BPH	The most predominant bacteria belonged to the following phyla; *Proteobacteria, Firmicutes, Actinobacteria, and Bacteroidetes* respectively. Among the viruses isolated, 41% were known tumorigenic viruses, including high-risk human papillomavirus (HPV 16&18), and human cytomegalovirus (HCMV)
Miyake M et al., 2019 [39]	45 prostate cancer and 33 BPH tissue specimens were screened for sexually transmitted infectious agents using PCR	*Mycoplasma genitalium* was the only organism independently associated with prostate cancer and with high Gleason scores
Shrestha E et al. 2018 [44]	Assessed the urinary microbiome of 135 men undergoing prostate needle biopsy	*Corynebacterium, Staphylococcus*, and *Streptococcus* species were the most predominant in both positive and negative biopsy cases. No species was significantly associated with prostate cancer.
Yu H et al. 2015 [45]	16S rRNA sequencing done on urine, seminal fluid, and expressed prostatic fluids (EPS) from men with BPH and Prostate cancer	*Bacteroidetes, Alphaproteobacteria, Firmicutes bacteria, Lachnospiraceae*, *Propionicimonas*, *Sphingomonas* spp. were significantly associated with Prostate cancer
Alanee S et al. 2018 [46]	Assessed the urinary and gut microbiota of 30 men undergoing trans-rectal prostate biopsy	A high abundance of *Veillonella, Streptococcus, and Bacteroides* spp. was found in Prostate cancer patients. No spp. was significantly associated with prostate cancer.
Liss MA et al. 2018 [57]	Assessed gut microbiota among 133 men undergoing trans-rectal prostate biopsy	There was significant differences in the microbial composition of the cancer and non-cancerous patients and non-significantly associated with Prostate cancer.
Golombos DM et al. 2018 [49]	Evaluated the gut microbiome of 20 men with BPH and prostate cancer	*A high abundance of Bacteroides massiliensis* in prostate cancer cases compared to BPH cases was found
Sfanos KS et al. 2018 [34]	Assessed microbiota of 30 healthy men and 30 men with localized, recurrent, and metastatic prostate cancer using 16S rDNA sequencing	There was a greater alpha diversity in the normal men compared to men with prostate cancer

## Conclusion

There is a growing body of knowledge concerning the relationship between the microbiome and prostate cancer. The current literature has shown that cancerous prostate tissue contains bacterial DNA, unlike healthy prostate tissue. Studies link urinary microbiome and chronic inflammation to prostate cancer risk and disease pathogenesis. Though not fully understood, studies indicate that chronic systemic inflammation and the immune system modulation are the mainstay mechanisms by which the gut microbiome propagates prostate cancer risk and pathogenesis. There is a lack of data on the role of the microbiome on prostate cancer therapies; however, current evidence shows that chemotherapy reduces gut microbiota diversity. Therefore there is a great need for more prospective studies to understand the exact mechanisms that elucidate the role of the microbiome in prostate cancer pathogenesis.

## Data Availability

Not applicable.
